# Characteristics of Hypervirulent *Klebsiella pneumoniae*: Does Low Expression of *rmpA* Contribute to the Absence of Hypervirulence?

**DOI:** 10.3389/fmicb.2020.00436

**Published:** 2020-03-17

**Authors:** Zhi-wei Lin, Jin-xin Zheng, Bing Bai, Guang-jian Xu, Fo-jun Lin, Zhong Chen, Xiang Sun, Di Qu, Zhi-jian Yu, Qi-wen Deng

**Affiliations:** ^1^Affiliated Shenzhen Sixth Hospital of Guangdong Medical University, Shenzhen, China; ^2^Key Laboratory of Medical Molecular Virology of Ministries of Education and Health, School of Basic Medical Science, Institutes of Biomedical Sciences, Shanghai Medical College of Fudan University, Shanghai, China

**Keywords:** *Klebsiella pneumoniae*, hypervirulence, extended-spectrum β-lactamases, carbapenem resistance, *rmpA*, aerobactin

## Abstract

Multidrug-resistant hypervirulent *Klebsiella pneumoniae* (MDR-hvKP) has been increasingly reported and is now recognized as a significant threat to public health; however, characterization of MDR-hvKP has not been systematically investigated. In the present study, 124 of 428 (28.92%) *K. pneumoniae* clinical isolates collected from January 2010 to December 2016 were identified with aerobactin and defined as hvKP; these included 94 non-MDR-KP, 20 extended-spectrum β-lactamase-producing *K. pneumoniae* (ESBL-KP), and 10 carbapenem-resistant *K. pneumoniae* (CR-KP) isolates. The remaining 304 isolates without presence of virulence factor aerobactin were defined as classic *K. pneumoniae* (cKP). The antimicrobial resistance rate of cKP was significantly higher than that of the hvKP isolates in the non-MDR-KP group, but showed no significant differences in the ESBL-KP and CR-KP groups. The detection frequencies of capsular serotype K1 (*magA*), hypermucoviscosity, sequence type 23 (ST23), and the virulence gene *rmpA* were significantly higher in the hvKP than cKP isolates in all three groups (*P* < 0.05). Most of the hypervirulent ESBL-KP and CR-KP isolates were K non-typeable (16/30) and harbored at least one gene for virulence (26/30). The hypervirulent ESBL-KP isolates primarily carried *bla*_*CTX–M*_ (12/20, 60%) genes, and the hypervirulent CR-KP isolates mainly carried *bla*_*NDM–*__1_ (8/10, 80%) genes. Moreover, three hypervirulent ESBL-KP and two hypervirulent CR-KP isolates showed resistance to tigecycline but were sensitive to colistin. The transcriptional levels of *rmpA* in cKP were much lower than that in hvKP isolates in all three groups. Furthermore, overexpression of *rmpA* in the *rmpA*-low-expression cKP isolates could enhance bacterial virulence in the mouse infection experiment. In conclusion, our data suggest that the capsular serotype K1 (*magA*), *rmpA*, hypermucoviscosity, and ST23 were strongly associated with hvKP in non-MDR-KP, ESBL-KP, and CR-KP groups, and low *rmpA* expression levels contributed to the absence of hypervirulent phenotype.

## Introduction

The hypervirulent *Klebsiella pneumoniae* (hvKP) was a new type of *K. pneumoniae* that caused several serious infections, such as pyogenic liver abscesses, meningitis, and endophthalmitis in young and healthy individuals (Struve et al., 2015). Since it was first reported in Taiwan in the mid-1980s (Liu et al., 1986), the hvKP infections have been reported around the world over the past few years (Decre et al., 2011; Siu et al., 2011; Chang et al., 2013). The hvKP and classic *K. pneumoniae* (cKP) strains can be distinguished by a combination of phenotypic and genotypic characteristics. Hypermucoviscosity, which is determined by a string test on agar plates, is a typical feature of hvKP strains and has been commonly used to define hvKP in previous studies (Li et al., 2014; Zhan et al., 2017). Most of the hvKP isolates belong to K1 and K2 capsular serotypes (Yeh et al., 2007). Several virulence genes, including regulator of mucoid phenotype A (*rmpA* and *rmpA2*), mucoviscosity-associated gene A (*magA*) and *wcaG* (encoding GDP-fucose synthetase) have been found to contribute to the hypervirulent phenotype (Lai et al., 2003; Yu et al., 2006; Hunt et al., 2011; Zhang R. et al., 2016). A recent study showed that aerobactin was a major virulence factor for the increased siderophore production in hvKP isolates and used for the definition of hvKP (Russo et al., 2014).

In recent years several studies have reported the existence and increasing incidence of multidrug-resistant hvKP (MDR-hvKP) isolates, especially extended-spectrum β-lactamase-producing hvKP (ESBL-hvKP) and carbapenem-resistant hvKP (CR-hvKP) (Yu et al., 2015b; Zhang et al., 2015; Zhang Y. et al., 2016; Zhan et al., 2017), which is now recognized as a serious threat to public health. However, the clinical and molecular characteristics of these MDR-hvKP isolates are not yet well understood. In this study, *K. pneumoniae* isolates carrying aerobactin were designated as hvKP and the differences in the distribution, molecular epidemiology, and clinical characteristics of hypervirulent determinants were investigated and compared among the non-MDR-KP, ESBL-KP, and CR-KP isolates. We also assessed the impact of *rmpA* expression level on virulence of cKP isolates and hypothesized that low levels of *rmpA* expression in cKP strains contributed to the absence of hypervirulent phenotype.

## Materials and Methods

### Bacterial Strains and Growth Conditions

A total of 428 non-duplicate *K. pneumoniae* strains were collected from inpatients at the Affiliated Shenzhen Sixth Hospital of Guangdong Medical University in China between January 2010 and December 2016. Bacterial species were identified by standard methods with a VITEK 2 compact system (Biomérieux, Marcy l’Etoile, France). All procedures performed were approved by the Ethical Committee of Shenzhen Nanshan People’s Hospital and were in accordance with the tenets of the 1964 Helsinki declaration and its later amendments. All strains were cultured in Luria-Bertani (LB; Oxoid, Basingstoke, United Kingdom) medium at 37°C with shaking at 220 rpm.

### Antimicrobial Susceptibility Testing and Phenotypic Confirmation of ESBL-KP and CR-KP

Antimicrobial susceptibility to several commonly used antibiotics such as amoxicillin/clavulanate, piperacillin/tazobactam, cefotaxime, ceftazidime, cefepime, ciprofloxacin, and amikacin were detected by broth microdilution in the VITEK 2 compact system. The minimal inhibitory concentrations (MICs) of tigecycline, colistin, imipenem, and meropenem were determined by the agar dilution method according to the guidelines of the Clinical and Laboratory Standards Institute (CLSI-M100-S27). The sensitivity results of antimicrobials were confirmed based on CLSI-M100-S27. ESBL production was detected by the agar dilution test with cefotaxime and ceftazidime, and CR-KP was confirmed by the modified Hodge test, both in accordance with the CLSI guidelines (CLSI-M100-S27). *Escherichia coli* ATCC 25922 was used as the quality control for all antimicrobial susceptibility tests.

### PCR Detection of Virulence-Associated Factors, β-Lactamase, and Carbapenemase Genes

Aerobactin positivity was determined by polymerase chain reaction (PCR) using the primers aerobactin-F/aerobactin-R and aerobactin-positive strains were designated as hvKP (Zhang Y. et al., 2016). *K*. *pneumoniae* virulence genes (*rmpA*, *rmpA2*, *magA*, and *wcaG*) and capsular serotypes (K1, K2, K5, K20, K54, and K57) were detected by PCR as previously described (Li et al., 2014; Candan and Aksoz, 2015; Yu et al., 2017). The extracted DNA of *K. pneumoniae* isolates served as templates for the PCR amplification. The *rmpA* amplicons were recovered for further sequencing. Mutations of *rmpA* in *K. pneumoniae* isolates were identified by comparing with the reference sequence of the *K. pneumoniae* NTUH-K2044 genotype (GenBank accession number: AP006725) (Wu et al., 2009). The β-lactamase genes *bla*_*TEM*_, *bla*_*SHV*_, *bla*_*CTX–M*_, and *bla*_*OXA–*__1_, and carbapenemase genes *bla*_*KPC*_, *bla*_*NDM–*__1_, *bla*_*OXA–*__48_, *bla*_*IMP*_, and *bla*_*VIM*_ were determined by PCR as previously described (Lewis et al., 2007; Candan and Aksoz, 2015). The primers used in this study are listed in Supplementary Table S1.

### String Test

The hypermucoviscous phenotype of *K. pneumoniae* isolates was confirmed by string test. The bacterial strains were grown on an agar plate at 37°C overnight and the formation of a mucoviscous string measuring >5 mm on a bacteriology inoculation loop was defined as a positive string test (Zheng et al., 2018a).

### Multilocus Sequence Typing (MLST) and Clonal Complex (CC)

Multilocus sequence typing (MLST) was conducted as previously described (Zheng et al., 2018a). Briefly, the housekeeping genes *gapA, infB, mdh, pgi, phoE, rpoB*, and *tonB* were amplified by PCR and sequenced. The sequence types (STs) were identified by the MLST database^1^. *Klebsiella pneumoniae* CCs were assigned using the phyloviz-2.0a program^2^. All primer sequences used in MLST are listed in Supplementary Table S1.

### Quantitative Real-Time (qRT)-PCR Analysis

The transcriptional levels of the virulence gene *rmpA* in hvKP and cKP isolates were analyzed by qRT-PCR. Sixteen hvKP (aerobactin positive and hypermucoviscous) and 13 cKP (aerobactin negative and non-hypermucoviscous) isolates were selected randomly from the non-MDR-KP, ESBL-KP, or CR-KP groups and grown to mid-log phase (4 h) in LB medium at 37°C. Total bacterial RNA was extracted using the RNeasy Mini Kit (Qiagen GmbH, Hilden, Germany). The extracted RNA was reverse transcribed into cDNA using the PrimeScript RT reagent kit (Takara Bio Inc., Shiga, Japan). The qRT-PCR was performed in a MasterCycler RealPlex System (Eppendorf, Hamburg, Germany) at the following amplification conditions: 95°C for 2 min, followed by 40 cycles of 5 s at 95°C and 34 s at 60°C. The housekeeping gene *rrsE* was used to normalize the expression of target genes. The transcriptional levels of *rmpA* were determined based on the 2^–ΔΔ*Ct*^ method and compared with that of *K. pneumoniae* NTUH-K2044 genotype. Each sample was carried out in triplicate. The primers used for qRT-PCR were designed based on the genome of *K. pneumoniae* NTUH-K2044, by using a Beacon designer software (Premier Biosoft International Ltd., Palo Alto, CA, United States) (Supplementary Table S1).

### The *rmpA* Overexpression Assay

The full-length of *rmpA* was amplified from the genomic DNA of NTUH-K2044. The PCR fragments were inserted into the plasmid pZP1137 with endonucleases *Nhe*I and *Bgl*II for gene overexpression (Zheng et al., 2018b). The positive clones were screened by kanamycin and verified by PCR and sequencing. The *rmpA* overexpression plasmid (p*rmpA*) were then introduced separately into three cKP isolates (LBKP61, EKP190, and CRKP16) with low expression levels of *rmpA* in qRT-PCR. The strains, plasmids, and primers used for *rmpA* overexpression are listed in Supplementary Tables S2, S3. The transcriptional level of *rmpA* was measured by qRT-PCR as described above. The overexpression of *rmpA* was induced with 1 mM arabinose (Ara).

### Intranasal Infection Model

The effect of *rmpA* overexpression on cKP isolates was determined by the mouse intranasal infection model as previously described (Palacios et al., 2018). Briefly, overnight bacterial cultures were diluted 1:200 in LB containing 1 mM arabinose and grown at 37°C until the optical density (OD) at 600 nm measured 1.0. Cells were harvested by centrifugation at 5,000 *g* for 5 min and resuspended in phosphate-buffered saline (PBS). Female C57BL/6 mice aged 5–7 weeks were anesthetized using a mixture containing ketamine (50 mg/kg) and xylazine (5 mg/kg) and were inoculated intranasally with 5 × 10^4^ colony forming unit (CFU)/mouse of each strain (*n* = 8 mice in each group). At 72 h post-inoculation, the mice were euthanized with pentobarbital (40 mg/kg). Lungs were removed, homogenized in PBS, serially diluted, and plated onto LB agar to quantify the CFU level. Results are reported as log10 CFU per gram of lung. Inoculation with PBS served as a negative control.

### Statistical Analysis

Experimental data were analyzed with Student’s *t*-test or one-way analysis of variance in IBM SPSS Statistics (version 20.0, Chicago, IBM, United States). Differences with a *P*-value < 0.05 were regarded statistically significant.

## Results

### Detection Frequency of hvKP in Non-MDR-KP, ESBL-KP, and CR-KP Isolates

A total of 428 non-repetitive *K. pneumoniae* clinical isolates were categorized into three groups: non-MDR-KP (205), ESBL-KP (181), and CR-KP (42) (Table 1). The detection frequency of hvKP (defined by aerobactin positivity) in all clinical isolates, non-MDR-KP, ESBL-KP, and CR-KP were 28.92% (124/428), 45.85% (94/205), 11.05% (20/181), and 23.81% (10/42), respectively. This indicates that the frequency of hvKP in ESBL-KP and CR-KP groups is significantly lower than that in non-MDR KP group (Table 1). Characteristics and comparison of the sample specimens’ origins among non-MDR-KP, ESBL-KP, and CR-KP groups are shown in Table 1.

**TABLE 1:** Distribution of hypervirulent (aerobactin positive) phenotype among non-MDR-KP, ESBL-KP, and CR-KP.

Isolates (*n*)	hvKP^b^ (*n* = 124)	cKP (*n* = 304)	*P*-value^c^
**Non-MDR-KP (205)**	94(45.85%)	111(54.15%)	< 0.0001(*vs*.*ESBL*-*KP*)
Sputum (66)	35(53.03%)	31(46.97%)	< 0.0001(*vs*.*sputumESBL*-*KP*)
Urine (23)	7(30.43%)	16(69.57%)	0.09(*vs*.*urineESBL*-*KP*)
Blood (94)	35(37.23%)	59(62.77%)	0.879(*vs*.*bloodESBL*-*KP*)
Abscess (15)	14(93.33%)	1(6.67%)	< 0.0001(*vs*.*othersESBL*-*KP*)
Others^a^ (7)	3(42.86%)	4(57.14%)	
**ESBL-KP (181)**	20(11.05%)	161(88.95%)	< 0.0001(*vs*.*non*-*MDR*-*KP*)
Sputum (110)	8(7.27%)	102(92.73%)	< 0.0001(*vs*.*sputumnon*-*MDR*-*KP*)
Urine (39)	5(12.82%)	34(87.18%)	0.09(*vs*.*urinenon*-*MDR*-*KP*)
Blood (17)	6(35.29%)	11(64.71%)	0.879(*vs*.*bloodnon*-*MDR*-*KP*)
Others (15)	1(6.67%)	14(93.33%)	
**CR-KP (42)**	10(23.81%)	32(76.19%)	0.008(*vs*.*non*-*MDR*-*KP*)
			0.29(*vs*.*ESBL*-*KP*)
Sputum (22)	5(22.73%)	17(77.27%)	0.013(*vs*.*sputumnon*-*MDR*-*KP*)
			0.026(*vs*.*sputumESBL*-*KP*)
Urine (4)	1(25.00%)	3(75.00%)	0.826(*vs*.*urinenon*-*MDR*-*KP*)
			0.503(*vs*.*urineESBL*-*KP*)
Blood (16)	4(25.00%)	12(75.00%)	0.344(*vs*.*bloodnon*-*MDR*-*KP*)
			0.52(*vs*.*bloodESBL*-*KP*)
Others (1)	0(0.00%)	1(100.00%)	

*^a^Others contain ascites, bile, pleural effusion, bronchoalveolar lavage fluid, and pericardial effusion. ^b^The hvKP were defined by aerobactin positivity. ^c^P value for hvKP among K. pneumoniae isolates.*

### Comparison of the Antimicrobial Susceptibility of hvKP and cKP Isolates in Non-MDR-KP, ESBL-KP, and CR-KP Groups

The antimicrobial susceptibilities of *K. pneumoniae* isolates are shown in Supplementary Table S4. Overall, the antimicrobial resistance rate of cKP was significantly higher than that of hvKP isolates in non-MDR-KP group but showed no differences in ESBL-KP and CR-KP groups. For example, 9.57% (9/94) of hvKP and 27.93% (31/111) of cKP isolates were resistant to ciprofloxacin in non-MDR-KP group while the hvKP and cKP isolates in ESBL-KP and CR-KP groups exhibited high-frequency resistance (resistance rate > 50%) to this antibiotic. The resistance rates of amikacin in hvKP and cKP isolates were less than 15% in non-MDR-KP and ESBL-KP groups but more than 30% in CR-KP group. Tigecycline susceptibility among non-MDR-KP, ESBL-KP, and CR-KP showed no significant difference; these isolates remained highly susceptible to tigecycline. Notably, colistin exhibited excellent antimicrobial activity against hvKP and cKP isolates in all three groups.

### Distribution of Virulence-Associated Factors in hvKP and cKP Isolates Among Non-MDR-KP, ESBL-KP, and CR-KP Groups

The distribution of virulence-associated factors of *K. pneumoniae* isolates are shown in Table 2. The capsular serotype K1 (*magA*) and virulence gene *rmpA* were more common in hvKP than cKP isolates in all three groups (*P* < 0.05). Further, K2, K5, K20, K54, and K57 were not significantly different between the hvKP and cKP isolates. The presence of *wcaG* and *rmpA2* were more common in hvKP isolates than cKP isolates in non-MDR-KP and ESBL-KP groups (*P* < 0.05), whereas they showed no difference between hvKP and cKP isolates in CR-KP group. The majority of hvKP isolates (97/124; 78.23%) while only 3.95% of the cKP (12/304) were hypermucoviscous (Table 2).

**TABLE 2:** Distribution of virulence factors among non-MDR-KP, ESBL-KP, and CR-KP isolates.

Characteristics	Non-MDR-KP (*n* = 205)			ESBL-KP (*n* = 181)			CR-KP (*n* = 42)		
	hvKP (*n* = 94)	cKP (*n* = 111)	*P*-value	hvKP (*n* = 20)	cKP (*n* = 161)	*P*-value	hvKP (*n* = 10)	cKP (*n* = 32)	*P*-value
**K serotype**									
K1	37(39.36%)	5(4.50%)	<0.001	6(30.00%)	12(7.45%)	0.001	3(30.00%)	2(6.25%)	0.043
K2	14(14.89%)	4(3.60%)	0.004	2(10.00%)	6(3.73%)	0.198	0(0.00%)	2(6.25%)	0.418
K5	1(1.06%)	0(0.00%)	0.276	0(0.00%)	0(0.00%)	/	0(0.00%)	0(0.00%)	/
K20	1(1.06%)	1(0.90%)	0.906	0(0.00%)	3(1.86%)	0.538	0(0.00%)	0(0.00%)	/
K54	6(6.38%)	6(5.41%)	0.766	1(5.00%)	0(0.00%)	0.004	0(0.00%)	3(9.38%)	0.315
K57	2(2.13%)	3(2.70%)	0.790	1(5.00%)	0(0.00%)	0.004	1(10.00%)	0(0.00%)	0.07
**Virulence gene**									
*wcaG*	12(12.77%)	6(5.41%)	0.064	3(15.00%)	5(3.11%)	0.015	0(0.00%)	0(0.00%)	/
*rmpA*	75(79.79%)	8(7.21%)	<0.001	18(90.00%)	4(2.48%)	<0.001	7(70.00%)	1(3.13%)	<0.001
*rmpA2*	26(27.66%)	5(4.50%)	<0.001	2(10.00%)	3(1.86%)	0.036	0(0.00%)	0(0.00%)	/
*magA*	37(39.36%)	5(4.50%)	<0.001	6(30.00%)	12(7.45%)	0.001	3(30.00%)	2(6.25%)	0.043
**MLST genotype**									
ST23	18(19.15%)	2(1.80%)	<0.001	5(25.00%)	1(0.62%)	<0.001	3(30.00%)	0(0.00%)	0.001
ST11	2(2.13%)	8(7.21%)	0.093	6(30.00%)	12(7.45%)	0.001	2(20.00%)	3(9.38%)	0.365
**Hypermucoviscosity**	73(77.66%)	7(6.31%)	<0.001	17(85.00%)	3(1.86%)	<0.001	7(70.00%)	2(6.25%)	<0.001

### Comparison of MLST Genotyping Between hvKP and cKP Isolates

**FIGURE 1:**
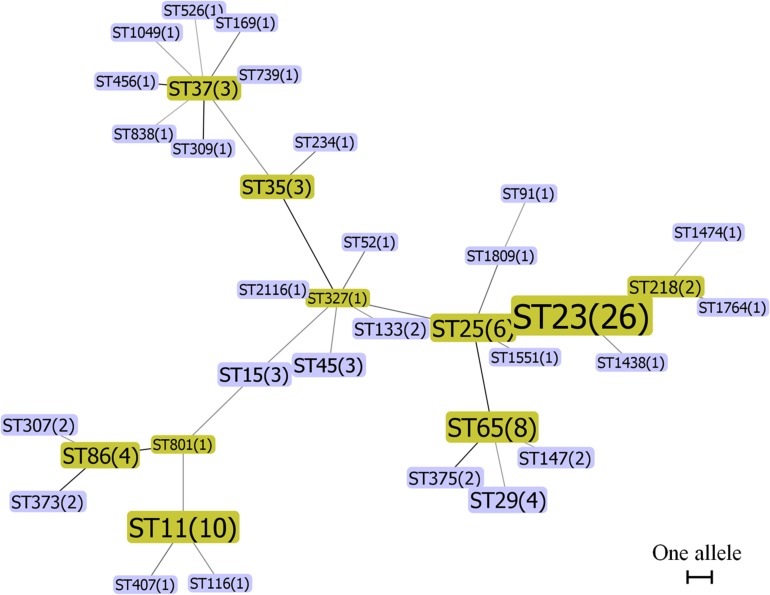
Minimum spanning tree of 102 hvKP isolates by MLST type and gene allele profile. Seven housekeeping genes (*gapA, infB, mdh, pgi, phoE, rpoB, and tonB*) were PCR-amplified and sequenced from all isolates according to the *Klebsiella pneumoniae* MLST protocol. Alleles and sequence types (STs) were assigned by the MLST database. *K. pneumoniae* CCs were identified by the phyloviz-2.0a program. Each node within the tree represented a single ST and the number of isolates. The size of the nodes was proportional to the number of isolates. Lines connecting each node indicate CCs. Length of lines between the node was proportional to the number of different alleles.

To compare the genetic relationship of hvKP and cKP isolates, the MLST genotyping and minimum spanning tree analysis were conducted. MLST enabled a clear sequence type (ST) assignment for 102 of the 124 hvKP isolates, in total 36 different STs. The most prevalent ST in the hvKP isolates was ST23 (*n* = 26, 25.49%), followed by ST11 (*n* = 10, 9.8%), ST65 (*n* = 8, 7.84%), and ST25 (*n* = 6, 5.88%). Two major MLST groups, ST23-like and ST317-like, were obtained based on minimum spanning tree analysis (Figure 1). The STs of 257 of 304 cKP isolates could be determined and were assigned to 88 different STs. ST37 was the most prevalent ST (*n* = 35, 13.62%), followed by ST15 (*n* = 31, 12.06%), ST11 (*n* = 23, 8.95%), and ST133 (*n* = 15, 5.84%). Minimum spanning tree analysis showed that all of the cKP isolates belonged to three major MLST groups, ST15-like, ST17-like, and ST25-like (Supplementary Figure S1). Sixteen *K. pneumoniae* isolates, including 6 hvKP and 10 cKP isolates, were identified with new allele code combinations (Supplementary Table S5). The STs of the remaining 53 isolates could not be determined due to the presence of one or more incomplete alleles. Moreover, the ST23 was more common in hvKP than cKP isolates in all three groups (*P* < 0.05, Table 2). These results indicated that ST23 was strongly associated with hvKP, while ST37 and ST15 were more common in the cKP isolates.

### Characteristics of the Hypervirulent ESBL-KP and CR-KP Isolates

Characteristics of the hypervirulent ESBL-KP and hypervirulent CR-KP isolates are shown in Table 3 (data of the hypervirulent non-MDR-KP isolates are listed in Supplementary Table S6). Among 20 hypervirulent ESBL-KP isolates, six were K1 capsular serotype and 10 were K non-typeable. Eighteen of these isolates harbored at least one virulence associated gene (*rmpA*, *rmpA2*, *magA*, or *wcaG*) in addition to aerobactin, and 17 isolates were hypermucoviscous. Six of them were ST11 and five, ST23. Twelve, seven, and two of the isolates carried *bla*_*CTX–M*_, *bla*_*TEM*_, and *bla*_*SHV*_ genes, respectively, encoding different ESBLs. Of the 10 hypervirulent CR-KP isolates, three were K1 and one was of K57 capsular serotype. Eight isolates harbored at least one gene for virulence (*rmpA* or *magA*). Seven isolates were hypermucoviscous. Three were ST23, two were ST11, two were ST25, and two were ST133. Eight isolates were New Delhi metallo-beta-lactamase 1 (NDM-1) positive and three were *K. pneumoniae* carbapenemase-2 (KPC-2) positive. Notably, three hypervirulent ESBL-KP and two hypervirulent CR-KP isolates were resistant to tigecycline but all of them were sensitive to colistin (Table 3).

**TABLE 3:** Molecular characteristics of the hypervirulent (aerobactin positive) ESBL-KP and CR-KP isolates.

Strain	Source	Capsule	Virulence genes	String-test	MLST	ESBL or carbapenemase types	Tigecycline susceptibility	Colistin susceptibility
**ESBL-KP (*n* = 20)**								
EKP3	Blood	K non-typeable	*rmpA*	+	ST15	CTX-M-14,	S	S
EKP16	Urine	K2	*rmpA, rmpA2*	+	ST65	CTX-M-14	S	S
EKP19	Sputum	K non-typeable		+	NT^*a*^	TEM-15	S	S
EKP29	Urine	K1	*rmpA, magA, wcaG*	+	ST23	CTX-M-15	S	S
EKP60	Urine	K non-typeable	*rmpA*	−	ST11	TEM-15, CTX-M-6	R	S
EKP85	Sputum	K non-typeable	*rmpA*	−	ST526	TEM-15	R	S
EKP91	Sputum	K1	*rmpA, magA*	+	ST23	CTX-M-15	S	S
EKP109	Urine	K1	*rmpA. magA*	+	ST23	CTX-M-14	S	S
EKP110	Blood	K54	*rmpA*	+	ST11	TEM-104	S	S
EKP120	Ascites	K1	*rmpA, magA*	+	ST11	TEM-15	S	S
EKP130	Sputum	K1	*rmpA, magA*	+	ST23	CTX-M-14	S	S
EKP138	Blood	K1	*rmpA, magA*	+	ST25	CTX-M-15	S	S
EKP141	Sputum	K57	*rmpA, wcaG*	+	ST11	SHV-12	S	S
EKP146	Blood	K non-typeable		+	NT	CTX-M-15	S	S
EKP162	Sputum	K non-typeable	*rmpA*	+	ST11	TEM-15	S	S
EKP169	Blood	K non-typeable	*rmpA*	−	ST1438	CTX-M-14	S	S
EKP170	Urine	K non-typeable	*rmpA*	+	ST11	CTX-M-18	R	S
EKP181	Sputum	K2	*rmpA*	+	ST37	CTX-M-15	S	S
EKP198	Blood	K non-typeable	*rmpA, rmpA2*	+	ST327	SHV-12	S	S
EKP204	Sputum	K non-typeable	*rmpA, wcaG*	+	ST23	TEM-48	S	S
**CR-KP (*n* = 10)**								
CRKP6	Blood	K non-typeable	*rmpA*	+	ST25	NDM-1	S	S
CRKP7	Blood	K1	*rmpA*, *magA*	+	ST23	NDM-1, KPC-2	S	S
CRKP10	Blood	K non-typeable	*rmpA*	+	ST11	NDM-1	R	S
CRKP14	Blood	K1	*rmpA, magA*	+	ST23	NDM-1	S	S
CRKP17	Sputum	K non-typeable		−	ST133	KPC-2	S	S
CRKP34	Sputum	K non-typeable	*rmpA*	+	ST23	NDM-1	S	S
CRKP36	Sputum	K1	*magA*	+	ST25	NDM-1	S	S
CRKP39	Sputum	K non-typeable	*rmpA*	+	ST11	NDM-1	R	S
CRKP40	Sputum	K non-typeable	*rmpA*	−	ST133	KPC-2	S	S
CRKP42	Urine	K57		−	NT	NDM-1	S	S

*^a^NT, not typeable.*

### Characteristics of *rmpA*-Positive cKP Isolates

**FIGURE 2:**
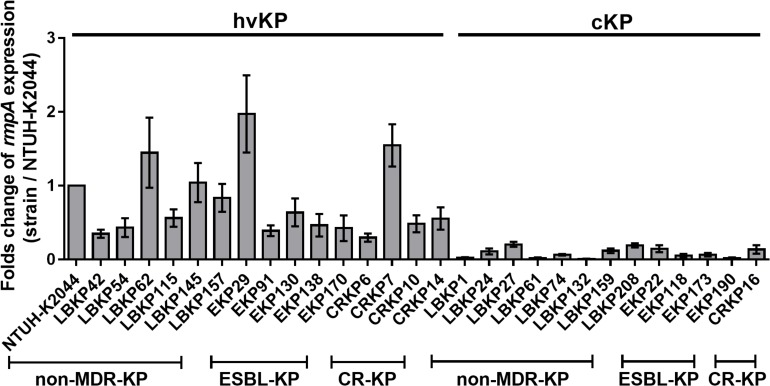
Relative gene expression of *rmpA* in 29 clinical isolates of *K. pneumoniae*. The hvKP and cKP isolates were cultured in LB for 4 h. Total RNA was extracted and transcriptional levels of *rmpA* were examined by qRT-PCR. The housekeeping gene *rrsE* was used as the endogenous reference gene. The *K. pneumoniae* NTUH-K2044 was used as the reference strain (transcriptional level = 1.0). All qRT-PCRs were carried out in triplicate. The hvKP isolates used in this assay were aerobactin positive and hypermucoviscous.

The *rmpA* gene was more frequently detected in hvKP than cKP isolates in all three groups (79.79% vs. 7.21%, *P* < 0.001, 90.00% vs. 2.48%, *P* < 0.001, 70% vs. 3.14%, *P* < 0.001, Table 2). This indicates an association of *rmpA* with hypervirulent phenotype (aerobactin positive) in *K. pneumoniae*. However, there were 13 *rmpA*-positive *K. pneumoniae* isolates (eight non-MDR-KP, four ESBL-KP, and one CR-KP) that did not show hypervirulence (aerobactin negative and non-hypermucoviscous). Owing to the high frequency of *rmpA* in hvKP isolates, the potential impact of *rmpA* on the virulence of cKP was investigated. First, genetic mutations in *rmpA* were determined. Among these 13 *rmpA*-positive strains, only two mutation sites of *rmpA* were found (LBKP27-K69I and EKP22-C102R), suggesting that gene polymorphism cannot entirely explain the phenotypic difference (hypervirulent or classic phenotype) caused by *rmpA* (Supplementary Table S7). Further, the difference in expression of *rmpA* between *rmpA*-positive hvKP (aerobactin positive, hypermucoviscous) and cKP (aerobactin negative, non-hypermucoviscous) isolates were compared by qRT-PCR. The transcriptional levels of *rmpA* in cKP isolates were much lower than those in hvKP isolates in all three groups (Figure 2), indicating that low expression of *rmpA* may lead to the absence of hypervirulent phenotype in cKP isolates.

### Overexpression of *rmpA* Enhances the Virulence of *rmpA*-Positive cKP Isolates

**FIGURE 3:**
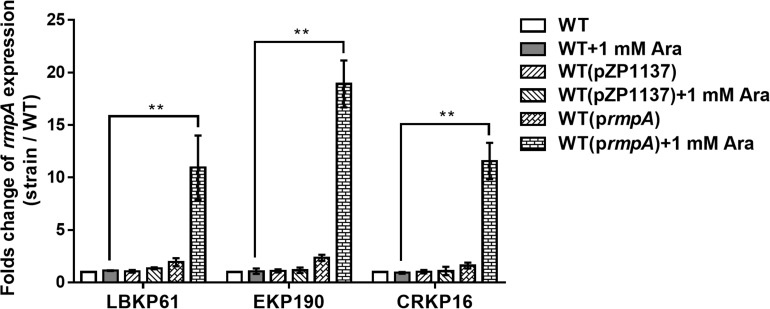
Relative transcriptional analysis of *rmpA* used in overexpression experiments. The *K. pneumoniae* strains were grown in LB for 4 h induced with or without 1 mM arabinose. Total RNA was extracted and transcriptional levels of *rmpA* were examined by qRT-PCR. The housekeeping gene *rrsE* was used as the endogenous reference gene. The clinical wild-type strains were used as the reference strain (transcriptional level = 1.0). All qRT-PCRs were carried out in triplicate. **P < 0.01.

**FIGURE 4:**
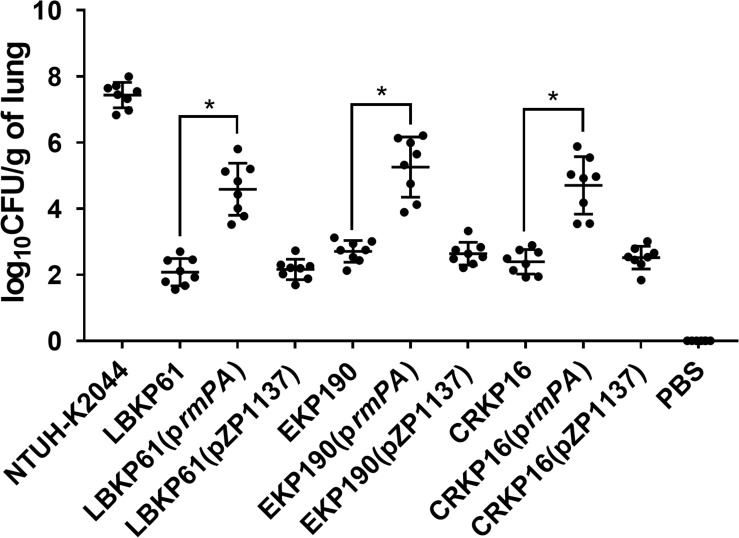
Effect of *rmpA* overexpression in *rmpA*-low-expression cKP isolates on virulence. Mice were inoculated intranasally with *K. pneumoniae* isolates. At 72 h post-inoculation, mice were euthanized, and lungs were homogenized and plated onto LB agar to quantify the CFU level. Each symbol represents one mouse. Mann–Whitney tests were performed for statistical analyses. *P < 0.05.

To confirm the impact of *rmpA* expression level on virulence in *rmpA*-positive cKP isolates, *rmpA* overexpressing plasmid was constructed and transformed into the *rmpA*-low-expression cKP isolates (LBKP61, EKP190, and CRKP16). The stable overexpression of *rmpA* in the transformed isolates was confirmed by qRT-PCR. The transcriptional level of *rmpA* in the transformed strains with 1 mM arabinose (Ara) induction increased 10.68–17.67-fold compared with that in the wild-type isolates (Figure 3). Further, the virulence of *rmpA* overexpression strains was determined by the mouse intranasal infection model. As showed in Figure 4, the colonized level of all three *rmpA* overexpression strains in the lung increased by more than two logs than that of the wild-type strains, indicating that overexpression of *rmpA* could enhance the virulence of the *rmpA*-low-expression cKP isolates.

## Discussion

Despite on-going research, there is still no consensus about the definition of hvKP. It is traditionally defined by hypermucoviscosity with a positive string test, but recent research has indicated that hypermucoviscosity and hypervirulence are two different phenotypes (Catalan-Najera et al., 2017). *K. pneumoniae* clinical isolates causing pyogenic liver abscesses without the characteristic hypermucoviscous phenotype have been found in an increasing number of studies (Luo et al., 2014; Cubero et al., 2016; Wu et al., 2017). These results suggested that the hypervirulent phenotype of *K. pneumoniae* is not dependent on hypermucoviscosity. Therefore, using hypermucoviscosity to define hvKP may not be accurate. The siderophore aerobactin is a major virulence factor in the progression of hvKP infection and survival in a human host (Russo et al., 2014, 2015). It is located on the large virulence plasmid (pLVPK) that is present in most hvKP isolates, but rarely present in cKP strains and considered as a genetic factor for both hypermucoviscous and hypervirulent phenotypes (Nassif and Sansonetti, 1986; Paczosa and Mecsas, 2016; Catalan-Najera et al., 2017). Because of the crucial role in hvKP, aerobactin positivity was considered a defining genetic trait for hvKP (Zhang Y. et al., 2016). In the present study, 45.85% of non-MDR-KP, 11.05% of ESBL-KP, and 23.81% of CR-KP isolates were defined as hvKP based on aerobactin positivity. Regarding hypermucoviscosity, 39.02, 11.05, and 21.43% of those isolates were identified as string test positivity in our study (Supplementary Table S8). Compared with the traditional designation criteria for hvKP by hypermucoviscosity, using aerobactin to define hvKP was more sensitive but less specific, which was similar to previous research findings (Zhang Y. et al., 2016). Considering that these two definitions of hvKP have a high degree of overlap (Supplementary Table S8), it may be more appropriate to combine aerobactin positivity and hypermucoviscosity when defining hvKP. Notably, hvKP isolates from abscesses contained more hypervirulent-associated factors compared to the hvKP isolates from other sources. All the 14 hvKP isolates from abscesses were *rmpA*-positive and hypermucoviscous. Eleven of them were K1 capsular serotype and nine were ST23 genotype (Supplementary Table S6).

Previous studies have shown that the *rmpA* gene, which is located on the large virulence plasmid pLVPK and participates in the enhancement of capsular production (Cheng et al., 2010), is a major virulence-associated factor in hvKP isolates (Yu et al., 2006; Zhang Y. et al., 2016). In our study, 80.65% (100/124) of hvKP isolates harbored *rmpA* gene while only 13 of 304 cKP isolates were *rmpA*-positive, indicating a strong association between *rmpA* and hypervirulence. Moreover, the potential impact of *rmpA* on virulence in those *rmpA*-positive cKP isolates was investigated. It has been reported that high tendency of *rmpA* mutations contributes to low virulence of *rmpA*-positive *K. pneumoniae* isolates (Yu et al., 2015a, b). We screened for *rmpA* genetic mutations in our *rmpA*-positive cKP strains, but did not identify any important mutation site. We further compared the expression levels of *rmpA* between hvKP and cKP isolates and found that the *rmpA* expression levels in cKP isolates were significantly lower than that in hvKP isolates in all three groups. Therefore, low expression of *rmpA* may lead to absence of the hypervirulent phenotype in cKP isolates. To confirm this assumption, a *rmpA* overexpressing plasmid was constructed and transformed into the *rmpA*-low-expression cKP isolates. We found that overexpression of *rmpA* could enhance the virulence of cKP isolates in the mouse model, which supported our hypothesis. However, the reason for the low expression of *rmpA* in cKP isolates is still unclear. A previous study showed that the expression of *rmpA* is regulated by the iron-responsive regulator Fur (Cheng et al., 2010), indicating that iron availability contributes to the expression of *rmpA*. Considering that aerobactin is one of the most important siderophores and all cKP isolates in our study were aerobactin-negative, the *rmpA* expression may depend on aerobactin, which needs to be further studied.

Besides *rmpA*, the capsular serotypes K1 and K2 have shown a strong correlation with hvKP in many reports (Zhao et al., 2016; Catalan-Najera et al., 2017; Guo et al., 2017). A previous study in Taiwan found that *K. pneumoniae* isolates with K1 or K2 capsular serotypes showed significantly higher virulence in the mouse model than isolates with other capsular serotypes (Yeh et al., 2007). In this study, the detection frequencies of hvKP isolates with K1 capsular serotype were 39.36% (37/94), 30% (6/20), and 30% (3/10) in non-MDR-KP, ESBL-KP, and CR-KP groups, respectively, which were significantly higher than that of cKP isolates. The proportion of K2 capsular serotype in hvKP and cKP isolates did not show a significant difference in our study. The virulence gene *magA* belongs to the K1 capsular operon and shows the same distribution pattern with K1 capsular serotype. ST23 was found to be the most prevalent ST in hvKP isolates and strongly correlated with the K1 capsular serotype in several previous studies (Siu et al., 2011; Qu et al., 2015; Guo et al., 2017). In this study, the ST23 genotype was more common in hvKP than cKP isolates in all three groups, suggesting the strong correlation between ST23 and hvKP. The proportion of ST23 in K1 capsular serotype hvKP isolates was 43.48% (20/46), which was lower than that reported in previous studies (Guo et al., 2017; Lee et al., 2017).

The hvKP strains are generally susceptible to commonly used clinical antimicrobial drugs (Paczosa and Mecsas, 2016). However, with the dissemination of genetic elements encoding ESBLs and carbapenemases, hypervirulent ESBL-KP and CR-KP isolates have been increasingly reported in the past few years (Yu et al., 2015b; Zhan et al., 2017; Liu and Guo, 2019). Previous studies showed that the identified CR-KP strains in China were mostly KPC-2-producer with ST11 or ST23 (Lee et al., 2017). In this study, 20 hypervirulent ESBL-KP and 10 hypervirulent CR-KP isolates were identified. Eight of them were ST11 and eight were ST23, implying the transmission of virulence genes to clonal lineages of cKP, e.g., ST11. The hypervirulent ESBL-KP isolates mainly carried *bla*_*CTX–M*_ (12/20) genes, which have been reported as the most common ESBL genotype in China (Yu et al., 2007). Notably, eight of 10 hypervirulent CR-KP isolates were NDM-1 type, which has rarely been reported in China before (Lee et al., 2016). Three hypervirulent ESBL-KP and two hypervirulent CR-KP isolates were resistant to tigecycline, which further reduced the therapeutic options for successful treatment of infection with these hypervirulent MDK-KP strains.

## Conclusion

Using the aerobactin positivity for defining hvKP in the present study revealed that the ESBL-KP and CR-KP isolates had significantly lower prevalence of hypervirulent phenotype but higher antibiotic resistance rates than the non-MDR-KP isolates. We also found that the capsular serotype K1 (*magA*), *rmpA*, hypermucoviscosity, and ST23 were strongly associated with hvKP in non-MDR-KP, ESBL-KP, and CR-KP groups. We showed that in addition to genetic mutations, low expression levels of *rmpA* also contributed to the hypervirulence-negative phenotype.

## Data Availability Statement

All datasets generated for this study are included in the article/Supplementary Material.

## Ethics Statement

All procedures performed were approved by the Ethical Committee of Shenzhen Nanshan People’s Hospital and were in accordance with the tenets of the 1964 Helsinki declaration and its later amendments.

## Author Contributions

ZL participated in the design of the study and drafted the manuscript. ZL, JZ, BB, and GX participated in antibiotic susceptibility test, string test, and detection of virulence factors, β-lactamase, and carbapenemase genes and participated in data analysis. FL and ZC performed the MLST and qRT-PCR assay. ZL and XS conducted the *rmpA* overexpression assay and mouse intranasal infection assay. DQ, ZY, and QD designed the study and assisted in revisions of the manuscript.

## Conflict of Interest

The authors declare that the research was conducted in the absence of any commercial or financial relationships that could be construed as a potential conflict of interest.

## Footnotes

1 https://bigsdb.pasteur.fr/klebsiella/

2 www.phyloviz.net

## Supplementary Material

FIGURE S1 Minimum spanning tree of 257 cKP isolates by MLST type and gene allele profile. Seven housekeeping genes (*gapA, infB, mdh, pgi, phoE, rpoB, and tonB*) were PCR-amplified and sequenced from all isolates according to the *K. pneumoniae* MLST protocol. Alleles and sequence types (STs) were assigned by the MLST database. *K. pneumoniae* CCs were identified by the phyloviz-2.0a program. Each node within the tree represented a single ST and the number of isolates. The size of the nodes was proportional to the number of isolates. Lines connecting each node indicated CCs. Length of lines between the node was proportional to the number of different alleles.

TABLE S1 Primers used in this study.

TABLE S2 Strains and plasmids used for the overexpression of *rmpA* in *Klebsiella pneumoniae*.

TABLE S3 Primers used for the overexpression of *rmpA* in *K. pneumoniae*.

TABLE S4 Distribution of antimicrobial resistance among non-MDR-KP, ESBL-KP, and CR-KP isolates.

TABLE S5 The allele information of 6 hvKP and 10 cKP isolates with new allele code combinations.

TABLE S6 Molecular characteristics of the hypervirulent non-MDR-KP isolates.

TABLE S7 The genotypes of *rmpA* among *K. pneumoniae* without hypervirulent phenotype.

TABLE S8 Comparison of the sensitivity and specificity of hvKP defined by aerobactin or hypermucoviscosity.
