# Supplementary figures and images for: Characteristics of Hypervirulent Klebsiella pneumoniae: Does Low Expression of rmpA Contribute to the Absence of Hypervirulence?
Source: Front Microbiol. 2020 Mar 17;11:436. doi: 10.3389/fmicb.2020.00436 (PMC7090111; doi:10.3389/fmicb.2020.00436)

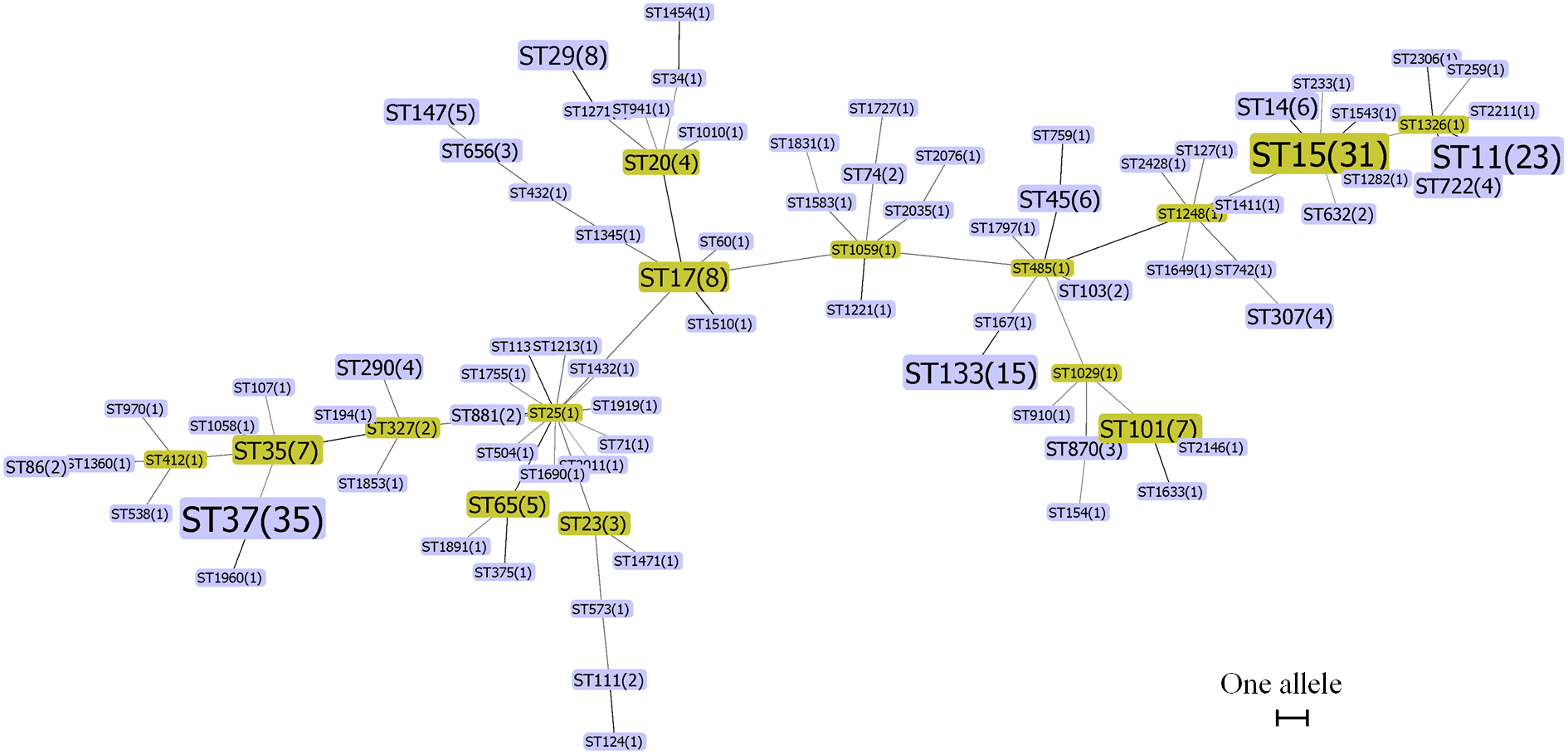

Supplement: FIGURE S1 — Minimum spanning tree of 257 cKP isolates by MLST type and gene allele profile. Seven housekeeping genes (gapA, infB, mdh, pgi, phoE, rpoB, and tonB) were PCR-amplified and sequenced from all isolates according to the K. pneumoniae MLST protocol. Alleles and sequence types (STs) were assigned by the MLST database. K. pneumoniae CCs were identified by the phyloviz-2.0a program. Each node within the tree represented a single ST and the number of isolates. The size of the nodes was proportional to the number of isolates. Lines connecting each node indicated CCs. Length of lines between the node was proportional to the number of different alleles. [file Image_1.TIF]
