# Supplementary material for: Characteristics of Hypervirulent Klebsiella pneumoniae: Does Low Expression of rmpA Contribute to the Absence of Hypervirulence?
Source: Front Microbiol. 2020 Mar 17;11:436. doi: 10.3389/fmicb.2020.00436 (PMC7090111; doi:10.3389/fmicb.2020.00436)
Supplement: TABLE S1 — Primers used in this study. [file Table_1.docx]

**TABLE S1****︱Primers used in this study.**

| **Target** | **Primer** | **Primer sequence (5'-3')** | **Amplicon size (bp)** | **Source** |
| --- | --- | --- | --- | --- |
| **Capsular serotypes** | | | | |
| K1 | K1-F | GTAGGTATTGCAAGCCATGC | 1045 | (Li et al., 2014) |
|  | K1-R | GCCCAGGTTAATGAATCCGT |  |  |
| K2 | K2-F | GACCCGATATTCATACTTGACAGAG | 641 | (Li et al., 2014) |
|  | K2-R | CCTGAAGTAAAATCGTAAATAGATGGC |  |  |
| K5 | K5-F | TGGTAGTGATGCTCGCGA | 280 | (Li et al., 2014) |
|  | K5-R | CCTGAACCCACCCCAATC |  |  |
| K20 | K20-F | CGGTGCTACAGTGCATCATT | 741 | (Li et al., 2014) |
|  | K20-R | GTTATACGATGCTCAGTCGC |  |  |
| K54 | K54-F | CATTAGCTCAGTGGTTGGCT | 881 | (Li et al., 2014) |
|  | K54-R | GCTTGACAAACACCATAGCAG |  |  |
| K57 | K57-F | CCAGTAATCAGTCCAGAAACAACC | 1037 | (Li et al., 2014) |
|  | K57-R | TAGCTTTTTTCATTCTTGTGTTTGTT |  |  |
| **Virulence genes** | | | | |
| *rmpA* | rmpA-F | ACTGGGCTACCTCTGCTTC | 516 | (Yu et al., 2017) |
|  | rmpA-R | CTTGCATGAGCCATCTTTCA |  |  |
| *rmpA2* | rmpA2-F | TGTGCAATAAGGATGTTACATTAGT | 610 | (Yu et al., 2017) |
|  | rmpA2-R | TTTGATGTGCACCATTTTTCA |  |  |
| *magA* | magA-F | GGTGCTCTTTACATCATTGC | 1283 | (Candan and Aksoz, 2015) |
|  | magA-R | GCAATGGCCATTTGCGTTAG |  |  |
| *wcaG* | wcaG-F | GGTTGGKTCAGCAATCGTA | 169 | (Candan and Aksoz, 2015) |
|  | wcaG-R | ACTATTCCGCCAACTTTTGC |  |  |
| Aerobactin | Aerobactin-F | GCATAGGCGGATACGAACAT | 556 | (Candan and Aksoz, 2015) |
|  | Aerobactin-R | CACAGGGCAATTGCTTACCT |  |  |
| **Detection of the mutation in *rmpA*** | | | | |
| *rmpA* | Full-rmpA-F | GTAACAAACGACGTTCAAGAG | 874 | This study |
|  | Full-rmpA-R | ATGTTCTGTGCGAGCGGAATA |  |  |
| **MLST diversity determination** | | | | |
| *gapA* | gapA-F | TGAAATATGACTCCACTCACGG | 662 | (Diancourt et al., 2005) |
|  | gapA-R | CTTCAGAAGCGGCTTTGATGGCTT |  |  |
| *infB* | infB-F | CTCGCTGCTGGACTATATTCG | 462 | (Diancourt et al., 2005) |
|  | infB-R | CTCGCTGCTGGACTATATTCG |  |  |
| *mdh* | mdh-F | CCCAACTCGCTTCAGGTTCAG | 756 | (Diancourt et al., 2005) |
|  | mdh-R | CCGTTTTTCCCCAGCAGCAG |  |  |
| *pgi* | pgi-F | GAGAAAAACCTGCCTGTACTGCTGGC | 566 | (Diancourt et al., 2005) |
|  | pgi-R | CGCGCCACGCTTTATAGCGGTTAAT |  |  |
| *phoE* | phoE-F | ACCTACCGCAACACCGACTTCTTCGG | 602 | (Diancourt et al., 2005) |
|  | phoE-R | TGATCAGAACTGGTAGGTGAT |  |  |
| *rpoB* | rpoB -F | GGCGAAATGGCWGAGAACCA | 1075 | (Diancourt et al., 2005) |
|  | rpoB -R | GAGTCTTCGAAGTTGTAACC |  |  |
| *tonB* | tonB-F | CTTTATACCTCGGTACATCAGGTT | 539 | (Diancourt et al., 2005) |
|  | tonB-R | ATTCGCCGGCTGRGCRGAGAG |  |  |
| **Detection of** **the β-lactamase and carbapenemase genes** | | | | |
| TEM | TEM-F | ATAAAATTCTTGAAGACGAAA |  | (Lewis et al., 2007) |
|  | TEM-R | GACAGTTACCAATGCTTAATCA |  |  |
| SHV | SHV-F | GGGTTATTCTTATTTGTCGC |  | (Lewis et al., 2007) |
|  | SHV-R | TTAGCGTTGCCAGTGCTC |  |  |
| CTX-M | CTX-F | AAAAATGATTGAAAGGTGGTTGT |  | (Lewis et al., 2007) |
|  | CTX-R | TTACAGCCCTTCGGCGATGA |  |  |
| OXA-1 | OXA-1F | CCAAAGACGTGG ATG |  | (Lewis et al., 2007) |
|  | OXA-1R | GTTAAATTCGACCCCAAG TT |  |  |
| KPC | KPC-F | CGTCTAGTTCTGCTGTCTTG | 798 | (Candan and Aksoz, 2015) |
|  | KPC-R | CTTGTCATCCTTGTTAGGCG |  |  |
| NDM-1 | NDM-1-F | GGTTTGGCGATCTGGTTTTC | 621 | (Candan and Aksoz, 2015) |
|  | NDM-1-R | CGGAATGGCTCATCACGATC |  |  |
| OXA-48 | OXA-48-F | GCGTGGTTAAGGATGAACAC | 438 | (Candan and Aksoz, 2015) |
|  | OXA-48-R | CATCAAGTTCAACCCAACCG |  |  |
| IMP | IMP-F | GGAATAGAGTGGCTTAAYTCTC | 232 | (Candan and Aksoz, 2015) |
|  | IMP-R | GGTTTAAYAAAACAACCACC |  |  |
| VIM | VIM-F | GATGGTGTTTGGTCGCATA | 390 | (Candan and Aksoz, 2015) |
|  | VIM-R | CGAATGCGCAGCACCAG |  |  |
| **Real-time PCR** | | | | |
| *rrsE* | qrrsE-F | CTACAATGGCATATACAA | 130 | This study |
|  | qrrsE-R | TTCTGATCTACGATTACT |  |  |
| *rmpA* | qrmpA-F | CCATTATCCACGGCTAAC | 134 | This study |
|  | qrmpA-R | TTGTTATCTTGCTTCGGTAG |  |  |

**TABLE S2︱Strains and plasmids used for** **the overexpression of *rmpA* in *K. pneumoniae*.**

| **Name** | **Description** | **Source** |
| --- | --- | --- |
| **strains** |  |  |
| NTUH-K2044 | Sequencing, *rmpA*- positive, aerobactin positivity and hypermucoviscosity | (Wu et al., 2009) |
| LBKP61 | Clinical isolate, *rmpA*- positive, aerobactin and hypermucoviscosity negative | This study |
| EKP190 | Clinical isolate, ESBL producing, *rmpA*-positive, aerobactin and hypermucoviscosity negative | This study |
| CRKP16 | Clinical isolate, carbapenem-resistant, *rmpA*-positive, aerobactin and hypermucoviscosity negative | This study |
| LBKP61(pZP1137) | LBKP61 introduced with plasmid pZP1137 | This study |
| EKP190(pZP1137) | EKP190 introduced with plasmid pZP1137 | This study |
| CRKP16(pZP1137) | CRKP16 introduced with plasmid pZP1137 | This study |
| LBKP61(p*rmpA*) | LBKP61 introduced with plasmid p*rmpA* | This study |
| EKP190(p*rmpA*) | EKP190 introduced with plasmid p*rmpA* | This study |
| CRKP16(p*rmpA*) | CRKP16 introduced with plasmid p*rmpA* | This study |
| **Plasmids** |  |  |
| pZP1137 | Medium copy number vector, myc tag, pBR322 ori, Arabinose-inducible, *kan*^R^ | (Zheng et al., 2018) |
| p*rmpA* | pZP1137 with insertion of the gene *rmpA*, for RmpA expression | This study |

**TABLE S3︱Primers used for the overexpression of *rmpA* in *K. pneumoniae*.**

| **Primers** | **Sequences（5’-3’）** | **Product**  **Length (bp)** | **Underline** |
| --- | --- | --- | --- |
| **Construction of the *rmpA* overexpression plasmids** | | | |
| OrmpA-F | CTAGCTAGCTGATGGATCAAAGTTACTGTT | 815 | NheI |
| OrmpA-R | GGAAGATCTATGTTCTGTGCGAGCGGAATA |  | BglII |
| **Verification of the *rmpA* overexpression plasmids** | | | |
| IDrmpA-F | CTATCATGCTACCGAAGCAAG | 872 |  |
| IDrmpA-R | ATCGATAGATTGTCGCACCTG |  |  |

**^a^**Underlined sequences represent the restriction enzyme sites of primers.

**TABLE S4︱****Distribution of antimicrobial resistance among non-MDR-KP, ESBL-KP and CR-KP isolates.**

| **Antimicrobial resistance** | **Non-MDR-KP (n=205)** | | | **ESBL-KP (n=181)** | | | **CR-KP (n=42)** | | |
| --- | --- | --- | --- | --- | --- | --- | --- | --- | --- |
|  | **hvKP (n=94)** | **cKP (n=111)** | ***P* value** | **hvKP (n=20)** | **cKP (n=161)** | ***P* value** | **hvKP (n=10)** | **cKP (n=32)** | ***P* value** |
| Amoxicillin / clavulanate | 18 (19.15%) | 55 (49.55%) | <0.001 | 14 (70.00%) | 103 (63.98%) | 0.595 | 9 (90.00%) | 29 (90.63%) | 0.953 |
| Piperacillin / tazobactam | 11 (11.70%) | 31 (27.93%) | 0.004 | 8 (40.00%) | 53 (32.92%) | 0.528 | 7 (70.00%) | 23 (71.88%) | 0.909 |
| Cefotaxime | 7 (7.45%) | 24 (21.62%) | 0.005 | 20 (100.00%) | 153 (95.03%) | 0.308 | 10 (100.00%) | 31 (96.88%) | 0.572 |
| Ceftazidime | 6 (6.38%) | 21 (18.92%) | 0.008 | 19 (95.00%) | 147 (91.30%) | 0.572 | 9 (90.00%) | 29 (90.61%) | 0.953 |
| Ciprofloxacin | 9 (9.57%) | 31 (27.93%) | 0.001 | 12 (60.00%) | 85 (52.80%) | 0.542 | 5 (50.00%) | 27 (84.38%) | 0.086 |
| Amikacin | 3 (3.19%) | 12 (10.81%) | 0.037 | 3 (15.00%) | 13 (8.07%) | 0.303 | 3 (30.00%) | 20 (62.50%) | 0.071 |
| Imipenem | 0 (0.00%) | 0 (0.00%) | / | 0 (0.00%) | 0 (0.00%) | / | 10 (100.00%) | 31 (96.88%) | 0.572 |
| Meropenem | 0 (0.00%) | 0 (0.00%) | / | 0 (0.00%) | 0 (0.00%) | / | 10 (100.00%) | 32 (100.00%) | / |
| Tigecycline | 3 (3.19%) | 17 (15.32%) | 0.004 | 3 (15.00%) | 22 (13.66%) | 0.870 | 2 (20.00%) | 5 (15.63%) | 0.746 |
| Colistin | 0 (0.00%) | 3 (2.70%) | 0.108 | 0 (0.00%) | 5 (3.11%) | 0.424 | 0 (0.00%) | 0 (0.00%) | / |

**TABLE S5︱The allele information of 6 hvKP and 10 cKP isolates with** **new allele code combinations.**

| **Isolate** | **Allele codes** | | | | | | | **New ST^a^** |
| --- | --- | --- | --- | --- | --- | --- | --- | --- |
|  | ***gapA*** | ***infB*** | ***mdh*** | ***pgi*** | ***phoE*** | ***rpoB*** | ***tonB*** |  |
| **hvKP (n=6)** | | | | | | | | |
| LBKP33 | 1 | 1 | 26 | 1 | 1 | 1 | 1 | 4694 |
| LBKP62 | 2 | 1 | 18 | 17 | 27 | 1 | 39 | 4696 |
| LBKP91 | 2 | 5 | 1 | 2 | 7 | 1 | 10 | 4698 |
| LBKP139 | 4 | 1 | 2 | 26 | 1 | 1 | 4 | 4700 |
| LBKP156 | 2 | 1 | 1 | 1 | 17 | 4 | 42 | 4701 |
| EKP146 | 2 | 1 | 2 | 1 | 9 | 4 | 26 | 4691 |
|  | | | | | | | | |
| LBKP17 | 2 | 3 | 2 | 1 | 9 | 1 | 38 | 4693 |
| LBKP57 | 18 | 67 | 1 | 63 | 142 | 38 | 169 | 4695 |
| LBKP86 | 4 | 6 | 5 | 1 | 1 | 28 | 34 | 4697 |
| LBKP105 | 2 | 1 | 2 | 1 | 9 | 4 | 16 | 4699 |
| EKP43 | 70 | 22 | 2 | 22 | 79 | 41 | 51 | 4687 |
| EKP64 | 2 | 1 | 2 | 2 | 10 | 4 | 10 | 4688 |
| EKP118 | 2 | 3 | 13 | 3 | 9 | 1 | 9 | 4689 |
| EKP127 | 4 | 1 | 5 | 1 | 3 | 44 | 4 | 4690 |
| EKP187 | 1 | 1 | 5 | 1 | 1 | 1 | 1 | 4692 |
| CRKP23 | 2 | 1 | 1 | 1 | 5 | 1 | 36 | 4686 |

^a^The data of these isolates were submitted to the *Klebsiella* MLST database (https://bigsdb.pasteur.fr/klebsiella/) and the new STs of them were designated by the database.

**TABLE S6︱Molecular characteristics of the hypervirulent non-MDR-KP isolates.**

| **Strain** | **Source** | **Capsule** | **Virulence genes** | **String-test** | **MLST** | **Tigecycline susceptibility** | **Colistin susceptibility** |
| --- | --- | --- | --- | --- | --- | --- | --- |
| LBKP11 | Abscess | K1 | *rmpA, magA* | + | ST23 | S | S |
| LBKP31 | Abscess | K57 | *rmpA* | + | ST23 | S | S |
| LBKP37 | Abscess | K1 | *rmpA, rmpA2, magA* | + | ST29 | S | S |
| LBKP54 | Abscess | K1 | *rmpA, magA* | + | ST23 | S | S |
| LBKP68 | Abscess | K1 | *rmpA, wcaG, magA* | + | ST23 | S | S |
| LBKP70 | Abscess | K2 | *rmpA, rmpA2* | + | ST29 | S | S |
| LBKP92 | Abscess | K1 | *rmpA, rmpA2, magA* | + | ST23 | S | S |
| LBKP108 | Abscess | K2 | *rmpA* | + | ST86 | S | S |
| LBKP119 | Abscess | K1 | *rmpA, rmpA2, magA* | + | ST23 | S | S |
| LBKP133 | Abscess | K1 | *rmpA, magA* | + | ST23 | S | S |
| LBKP146 | Abscess | K1 | *rmpA, rmpA2, magA* | + | ST23 | S | S |
| LBKP149 | Abscess | K1 | *rmpA, wcaG, magA* | + | ST373 | S | S |
| LBKP168 | Abscess | K1 | *rmpA, magA* | + | ST23 | S | S |
| LBKP174 | Abscess | K1 | *rmpA, rmpA2, magA* | + | ST739 | S | S |
| LBKP118 | Bile | K non-typeable | *rmpA* | + | ST147 | S | S |
| NOEKP115 | Bile | K non-typeable | *rmpA* | - | ST11 | S | S |
| LBKP9 | Blood | K non-typeable | *rmpA* | + | NT | S | S |
| LBKP14 | Blood | K54 | *rmpA* | + | ST65 | S | S |
| LBKP25 | Blood | K1 | *magA* | + | ST15 | S | S |
| LBKP33 | Blood | K non-typeable | *rmpA* | + | NT | S | S |
| LBKP39 | Blood | K2 | *rmpA* | - | ST147 | S | S |
| LBKP43 | Blood | K1 | *rmpA, rmpA2, magA* | + | ST23 | S | S |
| LBKP46 | Blood | K1 | *rmpA, magA* | + | NT | S | S |
| LBKP47 | Blood | K non-typeable | *rmpA, wcaG* | + | ST86 | S | S |
| LBKP62 | Blood | K1 | *rmpA, rmpA2, magA* | + | NT | S | S |
| LBKP72 | Blood | K5 | *rmpA* | + | ST45 | S | S |
| LBKP83 | Blood | K non-typeable |  | + | ST169 | S | S |
| LBKP84 | Blood | K1 | *rmpA, magA* | + | ST23 | S | S |
| LBKP95 | Blood | K non-typeable | *rmpA, rmpA2* | + | ST373 | S | S |
| LBKP101 | Blood | K2 | *rmpA, rmpA2* | - | ST1474 | S | S |
| LBKP115 | Blood | K1 | *rmpA, magA* | + | ST23 | S | S |
| LBKP121 | Blood | K2 | *rmpA* | + | ST65 | S | S |
| LBKP124 | Blood | K non-typeable | *wcaG* | - | ST407 | S | S |
| LBKP128 | Blood | K1 | *rmpA, magA* | + | NT | S | S |
| LBKP130 | Blood | K1 | *rmpA, rmpA2, magA* | + | ST29 | S | S |
| LBKP131 | Blood | K54 | *rmpA, wcaG* | - | ST23 | S | S |
| LBKP134 | Blood | K non-typeable | *rmpA, rmpA2* | + | ST86 | R | S |
| LBKP137 | Blood | K1 | *rmpA, rmpA2, magA* | + | ST23 | S | S |
| LBKP139 | Blood | K non-typeable | *rmpA* | + | NT | S | S |
| LBKP143 | Blood | K non-typeable | *wcaG* | - | ST23 | S | S |
| LBKP145 | Blood | K1 | *rmpA, magA* | + | ST65 | S | S |
| LBKP150 | Blood | K1 | *rmpA, rmpA2, magA* | + | ST1551 | S | S |
| LBKP153 | Blood | K2 | *rmpA, rmpA2* | + | ST52 | S | S |
| LBKP157 | Blood | K1 | *rmpA, magA* | + | NT | S | S |
| LBKP164 | Blood | K non-typeable |  | + | ST35 | S | S |
| LBKP167 | Blood | K54 | *rmpA* | - | ST375 | S | S |
| LBKP170 | Blood | K1 | *rmpA, magA* | + | ST86 | S | S |
| LBKP172 | Blood | K non-typeable |  | + | NT | S | S |
| LBKP175 | Blood | K1 | *rmpA, rmpA2, magA* | + | ST23 | S | S |
| LBKP184 | Blood | K1 | *rmpA, magA* | + | ST456 | S | S |
| LBKP187 | Blood | K2 | *rmpA* | + | ST65 | S | S |
| LBKP110 | Pleural effusion | K non-typeable | *wcaG* | - | ST91 | S | S |
| LBKP4 | Sputum | K non-typeable | *rmpA* | + | ST1764 | S | S |
| LBKP5 | Sputum | K1 | *rmpA, rmpA2, magA* | + | ST838 | S | S |
| LBKP7 | Sputum | K2 | *rmpA* | + | ST29 | S | S |
| LBKP28 | Sputum | K non-typeable |  | - | NT | S | S |
| LBKP42 | Sputum | K1 | *rmpA, magA* | + | ST65 | S | S |
| LBKP44 | Sputum | K non-typeable | *rmpA, rmpA2* | + | ST1809 | S | S |
| LBKP63 | Sputum | K1 | *rmpA, magA* | + | ST375 | S | S |
| LBKP76 | Sputum | K non-typeable | *wcaG* | + | ST35 | S | S |
| LBKP87 | Sputum | K54 | *rmpA* | - | NT | S | S |
| LBKP91 | Sputum | K non-typeable | *rmpA* | - | NT | S | S |
| LBKP99 | Sputum | K1 | *magA* | + | ST11 | S | S |
| LBKP113 | Sputum | K2 | *rmpA* | + | ST65 | R | S |
| LBKP114 | Sputum | K2 | *rmpA, rmpA2* | + | ST307 | S | S |
| LBKP123 | Sputum | K non-typeable |  | - | ST37 | S | S |
| LBKP127 | Sputum | K57 | *rmpA* | - | NT | S | S |
| LBKP135 | Sputum | K1 | *rmpA, rmpA2, wcaG, magA* | + | ST65 | S | S |
| LBKP141 | Sputum | K2 | *rmpA, rmpA2* | + | ST45 | S | S |
| LBKP156 | Sputum | K54 | *rmpA* | + | NT | S | S |
| LBKP163 | Sputum | K1 | *rmpA, rmpA2, magA* | + | ST234 | S | S |
| LBKP165 | Sputum | K20 |  | - | ST35 | S | S |
| LBKP171 | Sputum | K non-typeable | *rmpA* | + | ST116 | S | S |
| LBKP177 | Sputum | K1 | *rmpA, rmpA2, magA* | + | ST2116 | S | S |
| LBKP192 | Sputum | K2 | *rmpA* | + | NT | S | S |
| NOEKP1 | Sputum | K non-typeable |  | - | ST25 | S | S |
| NOEKP5 | Sputum | K non-typeable | *rmpA* | + | NT | S | S |
| NOEKP11 | Sputum | K54 | *rmpA, wcaG* | + | ST1049 | S | S |
| NOEKP34 | Sputum | K non-typeable | *wcaG* | - | ST25 | S | S |
| NOEKP36 | Sputum | K1 | *rmpA, magA* | + | ST801 | S | S |
| NOEKP51 | Sputum | K non-typeable | *rmpA* | - | ST23 | S | S |
| NOEKP80 | Sputum | K non-typeable | *rmpA* | + | ST15 | S | S |
| NOEKP117 | Sputum | K non-typeable | *rmpA, rmpA2* | + | ST309 | S | S |
| NOEKP133 | Sputum | K non-typeable |  | - | ST37 | R | S |
| NOEKP167 | Sputum | K non-typeable |  | + | ST45 | S | S |
| NOEKP186 | Sputum | K non-typeable | *rmpA* | + | ST11 | S | S |
| NOEKP215 | Sputum | K non-typeable | *rmpA* | - | NT | S | S |
| LBKP34 | Urine | K non-typeable | *rmpA* | - | NT | S | S |
| LBKP40 | Urine | K1 | *rmpA, rmpA2, magA* | + | ST218 | S | S |
| LBKP75 | Urine | K1 | *magA* | + | ST23 | S | S |
| LBKP88 | Urine | K2 | *rmpA* | + | ST218 | S | S |
| LBKP109 | Urine | K1 | *rmpA, rmpA2, magA* | + | NT | S | S |
| NOEKP76 | Urine | K2 | *wcaG* | + | ST25 | S | S |
| NOEKP140 | Urine | K non-typeable |  | - | NT | S | S |

**TABLE S7︱The genotypes of *rmpA* among *K. pneumoniae*** **without hypervirulent phenotype.**

| ***rmpA*-positive strains** | **Gene mutation** |
| --- | --- |
| LBKP1 | - |
| LBKP24 | - |
| LBKP27 | K69I |
| LBKP61 | - |
| LBKP74 | - |
| LBKP132 | - |
| LBKP159 | - |
| LBKP208 | - |
| EKP22 | C102R |
| EKP118 | *-* |
| EKP173 | *-* |
| EKP190 | *-* |
| CRKP16 | - |

**TABLE S8︱Comparison of the sensitivity and specificity of hvKP defined by aerobactin or hypermucoviscosity.**

| **Isolates (n)** | **Defined by aerobactin** | | **Defined by** **hypermucoviscosity** | | **Defined by aerobactin and hypermucoviscosity** | |
| --- | --- | --- | --- | --- | --- | --- |
|  | **hvKP (n=124)** | **cKP (n=304)** | **hvKP (n=109)** | **cKP (n=319)** | **hvKP (n=97)** | **cKP (n=331)** |
| **Non-MDR-KP (n=205)** | 94 (45.85%) | 111 (54.15%) | 80 (39.02%) | 125 (60.98%) | 73 (35.61%) | 132 (64.39%) |
| **ESBL-KP**  **(n=181)** | 20 (11.05%) | 161 (88.95%) | 20 (11.05%) | 161 (88.95%) | 17 (9.39%) | 164 (90.61%) |
| **CR-KP**  **(n=42)** | 10 (23.81%) | 32 (76.19%) | 9 (21.43%) | 33 (78.57%) | 7 (16.67%) | 35 (83.33%) |

**References**

Candan, E.D., and Aksoz, N. (2015). Klebsiella pneumoniae: characteristics of carbapenem resistance and virulence factors. *Acta Biochim Pol* 62(4)**,** 867-874. doi: 10.18388/abp.2015_1148.

Diancourt, L., Passet, V., Verhoef, J., Grimont, P.A., and Brisse, S. (2005). Multilocus sequence typing of Klebsiella pneumoniae nosocomial isolates. *J Clin Microbiol* 43(8)**,** 4178-4182. doi: 10.1128/JCM.43.8.4178-4182.2005.

Lewis, J.S., 2nd, Herrera, M., Wickes, B., Patterson, J.E., and Jorgensen, J.H. (2007). First report of the emergence of CTX-M-type extended-spectrum beta-lactamases (ESBLs) as the predominant ESBL isolated in a U.S. health care system. *Antimicrob Agents Chemother* 51(11)**,** 4015-4021. doi: 10.1128/AAC.00576-07.

Li, W., Sun, G., Yu, Y., Li, N., Chen, M., Jin, R., et al. (2014). Increasing occurrence of antimicrobial-resistant hypervirulent (hypermucoviscous) Klebsiella pneumoniae isolates in China. *Clin Infect Dis* 58(2)**,** 225-232. doi: 10.1093/cid/cit675.

Wu, K.M., Li, L.H., Yan, J.J., Tsao, N., Liao, T.L., Tsai, H.C., et al. (2009). Genome sequencing and comparative analysis of Klebsiella pneumoniae NTUH-K2044, a strain causing liver abscess and meningitis. *J Bacteriol* 191(14)**,** 4492-4501. doi: 10.1128/JB.00315-09.

Yu, W.L., Lee, M.F., Chen, C.C., Tang, H.J., Ho, C.H., and Chuang, Y.C. (2017). Impacts of Hypervirulence Determinants on Clinical Features and Outcomes of Bacteremia Caused by Extended-Spectrum beta-Lactamase-Producing Klebsiella pneumoniae. *Microb Drug Resist* 23(3)**,** 376-383. doi: 10.1089/mdr.2016.0018.

Zheng, J.X., Lin, Z.W., Sun, X., Lin, W.H., Chen, Z., Wu, Y., et al. (2018). Overexpression of OqxAB and MacAB efflux pumps contributes to eravacycline resistance and heteroresistance in clinical isolates of Klebsiella pneumoniae. *Emerg Microbes Infect* 7(1)**,** 139. doi: 10.1038/s41426-018-0141-y.
